# Association between arteriosclerosis, hemodynamic indices, and the risk of falls: receiver operating characteristic curve analysis for different indices in older individuals

**DOI:** 10.3389/fmed.2024.1469052

**Published:** 2024-10-04

**Authors:** Kexin Zhang, Yucen Ma, Di Yang, Mengyu Cao, Huijing Jin, Jiyan Leng

**Affiliations:** Department of Cadre Ward, The First Hospital of Jilin University, Changchun, China

**Keywords:** older adults, arteriosclerosis, hemodynamic, the risk of falls, comprehensive geriatric assessment

## Abstract

**Objective:**

This study aimed to assess the risk factors for falls and evaluate the correlation between arteriosclerosis, hemodynamic indices, and the risk of falls in older individuals.

**Method:**

This cross-sectional study included 920 individuals aged 60 and above from the cadre ward of the First Hospital of Jilin University. Data were obtained from the comprehensive geriatric assessment database of the cadre ward. Ankle-brachial indices (ABI) and brachial-ankle pulse wave velocity (baPWV) were measured using an OMRON arteriosclerosis detection device. Hemodynamic indices were assessed using the CSM3100 thoracic impedance hemodynamic detection system. Fall risk was evaluated with the fall risk assessment tool.

**Results:**

Significant differences in age, weight, education, smoking status, alcohol consumption, cognitive impairment, malnutrition, daily living abilities, depressive state, baPWV, ABI (all *p* < 0.001), systolic pressure, heart rate, cardiac stroke volume, and systemic vascular resistance were observed among the three groups (*p* = 0.011, *p* = 0.035, *p* = 0.005, *p* = 0.016). Ordinal logistic regression analysis indicated that the probability of an increase in fall risk by one level was 2.069 times higher for each unit decrease in educational background. Additionally, fall risk increased by 2.492 times for each additional year of age, 55.813 times for each unit of weight, 3.208 times for smoking status, 3.610 times for alcohol consumption, 4.665 times for cognitive impairment, 2.247 times for malnutrition, 2.596 times for ABI, 2.092 times for heart rate, and 1.586 times for cardiac stroke volume. The receiver operating characteristic curve analysis for fall risk in older individuals demonstrated that ABI was superior to heart rate and systemic vascular resistance in predicting the occurrence of falls.

**Conclusion:**

Our findings indicate that age, weight, educational background, smoking status, alcohol consumption, cognitive impairment, malnutrition, ABI, systolic blood pressure, heart rate, and cardiac stroke volume are associated with an increased risk of falls in older adults. Moreover, arteriosclerosis and hemodynamic parameters may aid in the early identification of fall risk among older individuals.

## 1 Introduction

Falls refer to a sudden and unconscious change in body position, resulting in a loss of balance and falling to the ground or a lower plane due to various risk factors (1). Annually, at least one-third of individuals aged 65 and above experience falls, with approximately 30% leading to physical injuries such as soft tissue damage, bleeding, fractures, and even death (2, 3). Falls are the leading cause of injury-related deaths among individuals aged 65 and older in China. According to the national disease surveillance system’s 2018 data, the fall-related death rate for this age group in China is 63.83 per 100,000, accounting for 39.72% of all injury-related deaths in this demographic. Recent studies have indicated that the incidence of falls increases with age (3, 4), posing a significant threat to the safety of older adults (5).

Older individuals experience an increased incidence of falls due to various intrinsic factors, including skeletal muscle degradation, an unstable center of gravity, impaired balance and coordination, reduced ability to maintain body posture, and decreased vision and cognitive response. Additionally, certain medical conditions such as arteriosclerosis, hypertension, cardiac insufficiency, arrhythmia, and valvular disease contribute to this risk. Vision impairments, gait instability, and blood pressure abnormalities also play a significant role. Furthermore, many medications can affect mental and emotional states, thereby increasing fall risk. Older hospitalized patients with cardiovascular diseases and hemodynamic issues are particularly prone to falls due to the degradation of physiological functions, the influence of underlying diseases, and the effects of medications. These falls significantly impact the physical and mental health of older patients and have become one of the primary contributors to mortality among older patients with cardiovascular disease. Arteriosclerosis (AS) is a systemic cardiovascular disease characterized by the accumulation of lipids, inflammation, cells, and tissue fibrosis within the arterial wall, leading to the formation of plaques and emboli. This condition is a root cause of most clinical cardiovascular diseases (CVD) (6). Previous studies have shown that frailty is a strong predictor of both short-term and long-term mortality in patients with severe acute myocardial infarction. Conversely, cardiovascular-related diseases, particularly hypertension, increase the risk of frailty in older adults (7–10). This paper aims to investigate the risk factors for falls in older individuals and explore the correlation between arteriosclerosis, hemodynamic indices, and fall risk in this population.

## 2 Materials and methods

### 2.1 Participants and eligibility criteria

Participants in this study were individuals aged 60 years and older who were admitted to the cadre ward of the First Hospital of Jilin University between May 1, 2019, and December 31, 2023. The inclusion criteria were as follows: (1) age ≥ 60 years and (2) completion of the comprehensive assessment questionnaire and older adult-specific tests. Exclusion criteria included: (1) incomplete basic data or missing indicators in the comprehensive assessment questionnaire and (2) the presence of malignant tumors, immune system disorders, severe liver or kidney dysfunction, or other serious illnesses.

### 2.2 Measurement

In this study, we analyzed the medical records of all individuals who met the eligibility criteria at the First Hospital of Jilin University between May 1, 2019, and December 31, 2023. Collected medical data included height, weight, sex, age, educational background, smoking history, and drinking history. Ankle-brachial index (ABI) and brachial-ankle pulse wave velocity (baPWV) were measured using an OMRON arteriosclerosis detection device. Hemodynamic parameters were evaluated using the CSM3100 thoracic impedance hemodynamic detection system. Furthermore, participants were assessed with the Activities of Daily Living Scale (ADL and IADL), Mini-Mental State Examination (MMSE), Geriatric Depression Scale (GDS), Falls Risk Assessment (FRA), and Mini Nutritional Assessment (MNA; Part I and Part II).

### 2.3 Arteriosclerosis test

A Japan-made Omron arteriosclerosis detection device was utilized, operated by trained medical personnel. Patients were instructed to remove their shoes and socks, turn their feet outward, and rest quietly in a supine position for 5 min. In accordance with the standard measurement protocol, cuffs were placed at the antecubital fossa and ankle of the upper limbs to detect the pulsation of the brachial artery and posterior tibial artery and measure systolic blood pressure. ECG clips were attached to the wrists, and a phonocardiogram sensor was positioned along the left sternal border at the fourth intercostal space to detect ECG signals, phonocardiograms, and pulse waveforms. Each measurement was performed twice, and the average values were recorded. The average ABI and baPWV were calculated based on the measurements from both the left and right sides (11, 12). Pasquale Mone et al. (13) found that P-Wave Dispersion (PWD), an electrocardiogram (ECG) parameter defined as the difference between the longest and shortest P-wave durations, was significantly correlated with MMSE scores. Increased arterial stiffness impairs the buffering capacity of large arteries, leading to elevated systolic blood pressure, reduced diastolic blood pressure, and widened pulse pressure. This elevation in afterload on the heart diminishes blood supply during diastole and increases the risk of arrhythmia. Previous studies have demonstrated a positive correlation between baPWV and PWD. Additionally, increased arterial stiffness is associated with the severity of cerebral small-vessel disease (SVD) and may serve as a predictor for incident dementia. As hypothesized, Sae Yamagishi et al. (14) investigated the predictive value of brachial-ankle pulse wave velocity (baPWV) for dementia and cognitive decline, and found that baPWV was indeed associated with both.

### 2.4 Hemodynamic test

The CSM3100 thoracic impedance hemodynamic detection system (Shenzhen Qianfan Electronics Co., Ltd.) was utilized to measure the patients’ hemodynamic parameters. Patients were instructed to lie flat and rest for at least 10 min. A blood pressure cuff was placed over the brachial artery pulsation site, and the skin of the neck and chest was disinfected with alcohol. Electrode sensors were attached to the bases of the left and right sides of the neck (below the earlobes) and along the midaxillary line (aligned with the sternal xiphoid process), with the larger head of the electrode facing proximally. The patients’ sex, weight, and height were recorded on the monitoring device. Thoracic electrical bioimpedance was measured by placing electrodes, and impedance changes were calculated through data processing to derive the hemodynamic parameters. Ge Tian et al. (15) investigated the causal relationship between higher blood pressure, pulse pressure, and an increased risk of frailty, suggesting that controlling hypertension may reduce the risk of frailty. Based on preliminary experimental results, we included indices such as stroke volume, systemic vascular resistance index, and thoracic fluid conductivity, given their potential relevance to the study’s objectives (16–19).

### 2.5 Fall risk assessment

The FRA tool was employed in this study. This scale, introduced in 2011 by the Ministry of Health of China (20), comprises 35 items, each scored from 1 to 3 points, with a maximum score of 53 points. Participants scoring ≤2 were classified as low risk, those scoring 3–9 as medium risk, and those scoring ≥10 as high risk. In 2014, Zhuge Yi et al. (21) referenced the revised Fall Efficacy Scale from abroad and applied the guideline scale to assess fall risk in the older adults (22). This scale has demonstrated high reliability and validity and is an effective tool for screening fall risk in older Chinese adults.

### 2.6 Other assessment

#### 2.6.1 Activities of daily living scale

The ADL and IADL scales assess the fundamental aspects of daily life (23). Both scales consist of 14 items, categorized into four levels: (1) Can perform independently, (2) Some difficulty, (3) Requires assistance, and (4) Unable to perform. The total score ranges from 14 to 56 points (24). These scales are further divided into two subscales: Physical Activities of Daily Living (PADL) and Instrumental Activities of Daily Living (IADL), each comprising seven items.

#### 2.6.2 Nutritional status

The MNA (25) has been widely used in prior studies to evaluate the nutritional status of older adults and serves as an evidence-based screening tool. An MNA score of less than 17 indicates malnutrition in participants.

#### 2.6.3 Depression

The Geriatric Depression Scale (GDS) (26) was specifically developed for older adults. The GDS consists of 30 items, each assigned one point, with a maximum possible score of 30. Participants scoring ≤10 were classified as normal, those scoring 11–20 were categorized as having mild depression, and those scoring 21–30 were classified as having moderate-to-severe depression (27).

#### 2.6.4 Cognitive function

The MMSE is a psychometric screening tool commonly used to evaluate cognitive function. The threshold for cognitive normality is determined by an individual’s educational background: a score greater than 20 for those with primary school education, and greater than 24 for those with junior high school education or higher. Scores below these thresholds indicate cognitive impairment. Previous research (28–30) has shown that, while the sensitivity of the MMSE is slightly lower than that of the MoCA in detecting mild cognitive impairment (MCI) and MCI prevalence is higher when using the MoCA compared to the MMSE, both tools demonstrate excellent specificity. Both the MMSE and MoCA consistently identify modifiable factors associated with MCI, providing valuable evidence for the development of intervention strategies. Gao Mingyue et al. (31) evaluated the factors influencing MMSE scores and the validity of its normative values for screening. Their findings demonstrated that the MMSE exhibits high reliability and validity, making it a suitable tool for screening cognitive impairment in older Chinese adults.

### 2.7 Statistical methods

Statistical analyses were conducted using SPSS statistical software (version 26.0; IBM Corp., Armonk, NY, United States). Continuous variables are presented as mean ± standard deviation (SD) or median (interquartile range), depending on their distribution. Categorical variables are expressed as percentages. Comparisons of quantitative data between two groups were performed using t-tests, while comparisons across multiple groups were made using one-way ANOVA. Categorical variables, such as sex, were compared using chi-square (χ^2^) tests. If the data did not follow a normal distribution, rank sum tests were applied. Ordinal logistic regression analysis was employed to examine the correlations between arteriosclerosis, hemodynamic indices, and fall risk. The area under the curve (AUC) was calculated by plotting the receiver operating characteristic (ROC) curve to assess the association between various indices and fall risk. The Youden index was applied to determine the optimal cutoff point. All statistical tests were two-sided, with *p* < 0.05 considered statistically significant.

## 3 Results

### 3.1 Baseline characteristics

As presented in Table 1, the study included a total of 920 participants. Statistically significant differences were observed among the three groups regarding age, weight, education level, smoking status, alcohol consumption, cognitive impairment, malnutrition, activities of daily living, and depressive state (all *p* < 0.001).

**Table 1:** Comparison of demographic information of the individuals.

	All	Low risk group (*N* = 212)	Medium risk group (*N* = 358)	High risk group (*N* = 350)	*p*
Age (years)	75.1 ± 10.2	70.9 ± 9.1	76.0 ± 9.6^a^	76.7 ± 10.9^a^	<0.001^*^
Gender					0.159
Male	615 (66.8%)	146 (68.9%)	226 (63.1%)	243 (69.4%)	
Female	305 (33.2%)	66 (31.1%)	132 (36.9%)	107 (30.6%)	
Height (cm)	166.4 ± 7.8	167.3 ± 7.3	165.8 ± 8.1^a^	166.4 ± 7.8	0.072
Weight (kg)	68.0 ± 11.5	71.2 ± 10.6	66.8 ± 12.0^a^	67.2 ± 11.2^a^	<0.001^*^
Education					<0.001^*^
Illiteracy	111 (12.1%)	14 (6.6%)	22 (6.1%)	75 (21.4%)	
Primary School	277 (30.1%)	43 (20.3%)	81 (22.6%)	153 (43.7%)	
Middle School	25 (2.7%)	3 (1.4%)	17 (4.7%)	5 (1.4%)	
College Degree and Above	507 (55.1%)	152 (71.6%)	238 (66.5%)	117 (33.4%)	
Smoking					<0.001^*^
Yes	620 (67.5%)	169 (79.7%)	277 (77.9%)	174 (49.7%)	
No	298 (32.5%)	43 (20.3%)	79 (22.2%)	176 (50.3%)	
Alcohol					<0.001^*^
Yes	559 (60.8%)	152 (71.7%)	263 (73.5%)	144 (41.2%)	
No	361 (39.2%)	60 (28.3%)	95 (26.5%)	206 (58.9%)	
Cognitive Impairment					<0.001^*^
Yes	85 (9.2%)	4 (1.9%)	21 (5.9%)	60 (17.1%)	
No	835 (90.8%)	208 (98.1%)	337 (94.1%)	290 (82.9%)	
Malnutrition					<0.001^*^
Yes	177 (19.2%)	12 (5.7%)	76 (21.2%)	89 (25.4%)	
No	743 (80.8%)	200 (94.3%)	282 (78.8%)	261 (74.6%)	
Ability of Daily Living (score)	16.9 ± 5.6	14.5 ± 1.7	15.6 ± 3.6^a^	19.7 ± 7.4^a^^,^^b^	<0.001^*^
Depressive State (score)	18.5 ± 12.9	21.2 ± 9.3	22.1 ± 10.6	32.4 ± 9.5^a^^,^^b^	<0.001^*^

a indicates that the difference is statistically significant compared with the low-risk group.

b indicates that the difference is statistically significant compared with the medium-risk group.

*p < 0.05.

### 3.2 Comparison of arteriosclerosis and hemodynamic indices among low-, medium-, and high-risk group

As presented in Table 2, significant differences were observed among the three groups regarding baPWV, ABI, systolic blood pressure, heart rate, cardiac stroke volume, and systemic vascular resistance (*p* < 0.05). The high-risk group exhibited higher baPWV, systolic blood pressure, and systemic vascular resistance, as well as lower ABI, heart rate, and cardiac stroke volume compared to the other two groups. All of these differences were statistically significant (*p* < 0.05).

**Table 2:** Comparison of arteriosclerosis and hemodynamic indices among the low-, medium-, and high-risk groups.

	All	Low risk group	Medium risk group	High risk group	*p*
baPWV (right)(cm/s)	1766.5 ± 398.3	1632.7 ± 276.5	1802.1 ± 369.2^a^	1811.3 ± 466.8^a^	<0.001^*^
baPWV (left)(cm/s)	1766.8 ± 391.5	1631.6 ± 263.7	1784.6 ± 353.0^a^	1830.6 ± 467.7^a^	<0.001^*^
baPWV (cm/s)	1766.3 ± 366.7	1632.2 ± 263.4	1792.6 ± 344.3^a^	1820.6 ± 419.8^a^	<0.001*
ABI (right)	1.09 ± 0.15	1.13 ± 0.13	1.11 ± 0.14	1.05 ± 0.17^a^^,^^b^	<0.001^*^
ABI (left)	1.10 ± 0.45	1.12 ± 0.15	1.10 ± 0.14	1.10 ± 0.71	0.100
ABI	1.10 ± 0.26	1.12 ± 0.13	1.10 ± 0.13	1.08 ± 0.38^a^	0.011^*^
Systolic Pressure (mmHg)	138.1 ± 19.1	136.1 ± 17.8	138.7 ± 20.2^a^	138.8 ± 18.8^a^	0.035^*^
Diastolic Pressure (mmHg)	77.4 ± 11.2	78.7 ± 10.7	77.1 ± 11.3	76.8 ± 11.3^a^	0.475
Heart Rate (cpm)	72.5 ± 10.0	74.2 ± 10.0	72.6 ± 10.6	71.4 ± 9.1^a^	0.005^*^
Cardiac Stroke Volume (ml)	81.0 ± 22.3	79.7 ± 20.5	83.7 ± 21.6^a^	79.1 ± 23.8^b^	0.016^*^
Thoracic Fluid Conductivity (/kohm)	0.03 ± 0.07	0.03 ± 0.06	0.03 ± 0.07	0.03 ± 0.08	0.053
Systemic Vascular Resistance (Dyne sec/cm^−5^ m^2^)	156.1 ± 55.3	159.8 ± 60.2	148.5 ± 42.9^a^	161.7 ± 62.2^b^	0.003^*^

baPWV, brachial-ankle pulse wave velocity. ABI, ankle-brachial indices.

a indicates that the difference is statistically significant compared with the low-risk group.

b indicates that the difference is statistically significant compared with the medium-risk group.

*p < 0.05.

### 3.3 Ordinal logistic regression analysis of body composition and serological indices of the low-, medium-, and high-risk groups to predict the risk of falls

As shown in Table 3, the probability of fall risk increasing by one level was 2.069 times higher for each unit decrease in educational background (OR = 0.483, 95% CI: 0.862–3.333, *p* = 0.006). Furthermore, the probability of fall risk increasing by one level was 2.492, 55.813, 4.665, 2.596, 2.092, and 1.586 times higher for each additional unit of age, weight, cognitive impairment, ABI, heart rate, and cardiac stroke volume, respectively (OR = 2.492, 95% CI: 0.862–3.333, *p* < 0.001; OR = 55.813, 95% CI: 3.305–942.250, *p* = 0.005; OR = 4.665, 95% CI: 2.875–7.576, *p* < 0.001; OR = 2.596, 95% CI: 1.895–3.557, *p* < 0.001; OR = 2.092, 95% CI: 1.404–3.117, *p* < 0.001; OR = 1.586, 95% CI: 1.118–2.249, *p* = 0.010).

**Table 3:** Ordinal logistic regression analysis of arteriosclerosis and hemodynamic indices to predict the risk of falls among older adults in the low-, medium-, and high-risk groups.

Indicators	B	SE	Wald	OR	95%CI	*p*
Age (year)	0.913	0.148	37.841	2.492	0.862–3.333	<0.001^*^
Weight (kg)	4.022	1.442	7.778	55.813	3.305–942.250	0.005^*^
Education	−0.727	0.201	13.033	0.483	0.326–0.717	0.006^*^
Cognitive Impairment (Yes)	1.540	0.247	38.895	4.665	2.875–7.576	<0.001^*^
baPWV (cm/s)	0.050	0.183	0.075	1.051	0.734–1.505	0.784
ABI	0.954	0.161	35.164	2.596	1.895–3.557	<0.001^*^
Systolic Pressure (mmHg)	0.182	0.123	2.180	1.200	0.942–1.528	0.140
Heart Rate (cpm)	0.738	0.203	13.169	2.092	1.404–3.117	<0.001^*^
Cardiac Stroke Volume (ml)	0.461	0.178	6.687	1.586	1.118–2.249	0.010^*^
Systemic Vascular Resistance (Dyne sec/cm^−5^ m^2^)	0.292	0.157	3.449	1.339	0.984–1.822	0.063

Dependent Variable: the risk of falls. Independent Variable: age, weight, education, cognitive impairment, baPWV, ABI, systolic pressure, heart rate, cardiac stroke volume, systemic vascular resistance. baPWV, brachial-ankle pulse wave velocity. ABI, ankle-brachial indices. *p < 0.05.

### 3.4 The association of different indices with the occurrence of falls

**Figure 1:**
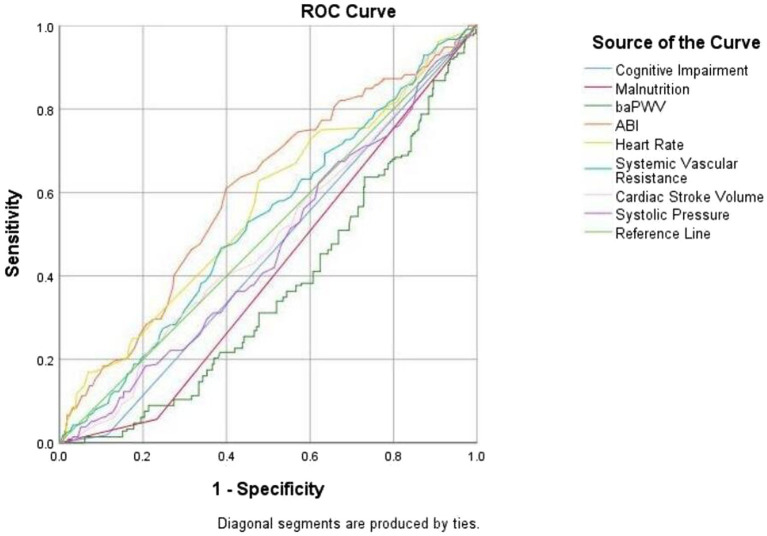
The receiver operating characteristics curve of different indices for the occurrence of falls.

The correlation between various indices and the occurrence of falls in older adults was analyzed and compared. As shown in Table 4, the area under the curve (AUC) for ABI, heart rate, and systemic vascular resistance was greater than 0.5, with AUC values of 0.602, 0.565, and 0.529, and corresponding Youden’s Indices of 21.02, 15.00, and 8.00, respectively. The analysis indicated that ABI was superior in predicting the occurrence of falls compared to heart rate and systemic vascular resistance. The corresponding ROC curve is displayed in Figure 1.

**Table 4:** Correlation between different indices and the occurrence of falls.

	AUC (95%CI)	*p*	Youden’s index (%)	Sensitivity (%)	Specificity (%)
Cognitive Impairment (Yes)	0.452 (0.410–0.494)	0.035^*^	9.55	98.12	11.43
Malnutrition (Yes)	0.412 (0.371–0.452)	<0.001^*^	17.64	94.31	23.33
baPWV (cm/s)	0.365 (0.325–0.405)	<0.001^*^	0.47	97.63	2.84
ABI	0.602 (0.559–0.645)	<0.001^*^	21.02	60.81	60.21
Heart Rate (cpm)	0.565 (0.520–0.610)	0.004^*^	15.00	62.70	52.30
Systolic Pressure (mmHg)	0.464 (0.421–0.507)	0.111	1.12	97.21	4.01
Cardiac Stroke Volume (ml)	0.488 (0.445–0.532)	0.602	4.37	92.93	11.44
Systemic Vascular Resistance (Dyne sec/cm^−5^ m^2^)	0.529 (0.485–0.572)	0.207	8.00	46.70	61.30

baPWV, brachial-ankle pulse wave velocity. ABI, ankle-brachial indices. *p < 0.05.

## 4 Discussion

**Figure 2:**
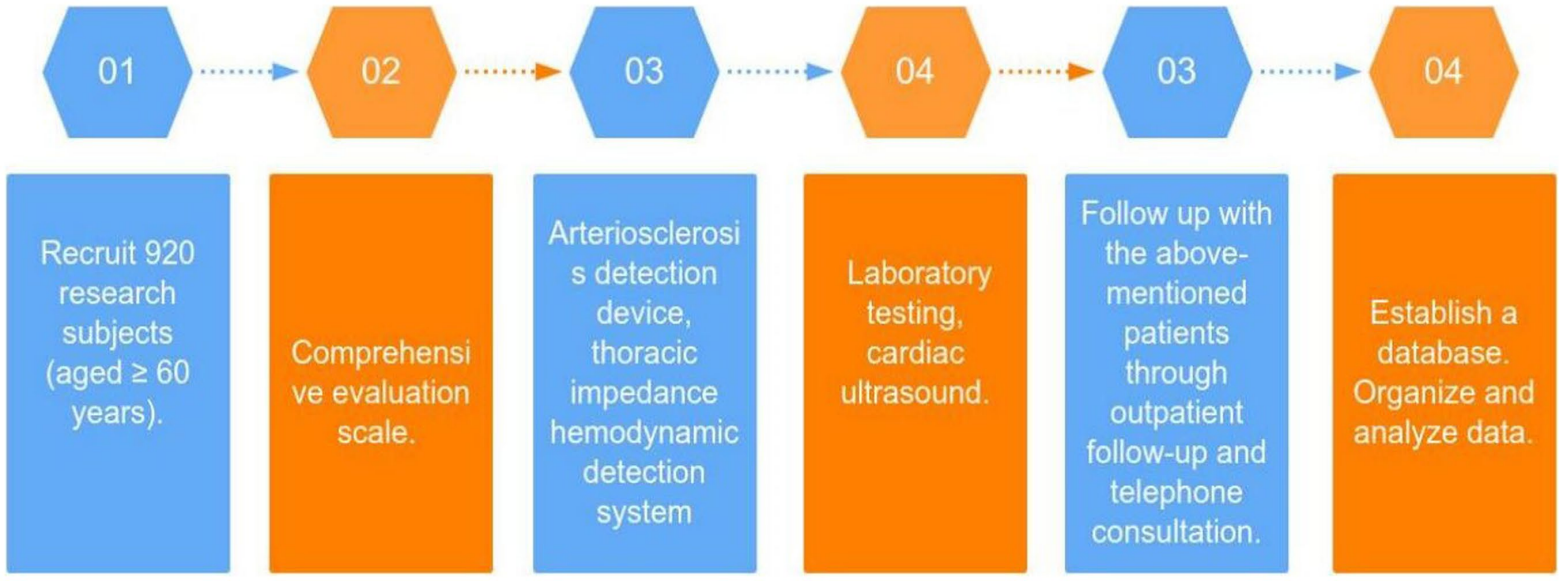
Study flow chart.

Our study found that increased baPWV, systolic blood pressure, and systemic vascular resistance, along with decreased ABI, heart rate, and cardiac stroke volume, are significant risk factors for falls in older adults. Falls, a common and serious aspect of geriatric syndromes, are influenced by multiple factors, either independently or in combination. Preclinical cardiovascular disease (CVD) is also widespread among older adults. Recent treatment guidelines for CVD risk factors, such as hypertension, hypercholesterolemia, and procoagulant conditions (e.g., atrial fibrillation), have been expanded to include a larger proportion of older individuals (32–34). There is growing evidence suggesting a correlation between arteriosclerosis, hemodynamic abnormalities, and falls in older adults (Figure 2).

Ge Tian et al. (15) investigated the causal association between elevated blood pressure, pulse pressure, and an increased risk of frailty. Since arteriosclerosis and hemodynamic changes contribute to greater frailty and muscle weakness, which in turn reduce mobility, effectively managing these conditions can help mitigate the risk of falls.

Patients with cardiac insufficiency are prone to falls due to reduced cerebral perfusion, decreased cardiac output, unstable posture, and other symptoms during acute episodes (35, 36). Cardiovascular disease is prevalent among older adults (37, 38) and is linked to an elevated risk of falls (39–41). Left untreated, cardiovascular disease may theoretically increase the risk of falls. The cardiovascular system plays several critical roles in bodily function, such as ensuring an adequate blood supply to peripheral muscles during activity and maintaining the blood pressure required for cerebral perfusion to support balance (42). However, treatments aimed at preventing cardiovascular events in older adults, such as the initiation of antihypertensive medication, are associated with a heightened risk of falls in the short term. Arteriosclerosis, the primary cause of cardiovascular disease in older adults, is also closely linked to the risk of falls in this population (43, 44).

Studies have shown that subclinical cardiovascular disease increases the risk of falls. The causes of falls are multifactorial and include muscle weakness, impaired physical function during transitions (e.g., moving from sitting to standing or walking), and postural imbalance while standing (45, 46). The cardiovascular system plays a critical role in these basic functions, including increasing heart rate during positional changes, maintaining peripheral blood supply, and stabilizing blood pressure to counteract the effects of gravity while standing. These functions are essential for maintaining balance, which is mediated by the central nervous system (42, 47). Thus, functional pathway defects leading to falls may originate from cardiovascular conditions, even before these conditions present as clinical cardiovascular disease. The findings of this study support these conclusions.

This study found that fall risk is closely associated with age, education level, cognitive impairment, and daily living ability. Although falls can occur in any age group, the risk is significantly higher in older adults compared to younger individuals (48, 49). Our research suggests that fall occurrence is related to education level, potentially due to the stronger health awareness among individuals with higher educational attainment. Numerous studies have shown that older adults with cognitive impairment are more prone to falling (50, 51), likely because their ability to process and respond to external information is diminished, increasing the likelihood of falls.

Our research suggests that older adults should regularly monitor arteriosclerosis and hemodynamic parameters, including ABI, heart rate, and systemic vascular resistance. Assessing the extent of atherosclerosis, screening for cardiovascular diseases, and implementing early detection and prevention strategies for falls are essential for improving the health outcomes of older adults.

## 5 Conclusion

Our findings indicate that age, weight, educational background, smoking status, alcohol consumption, cognitive impairment, malnutrition, ABI, systolic blood pressure, heart rate, and cardiac stroke volume are associated with an increased risk of falls in older adults. Moreover, arteriosclerosis and hemodynamic parameters may aid in the early identification of fall risk among older individuals.

## Data Availability

The original contributions presented in the study are included in the article/supplementary material, further inquiries can be directed to the corresponding author.
